# Sterility in Woman—Its Ætiology and Treatment

**Published:** 1891-08

**Authors:** E. S. McKee

**Affiliations:** Cincinnati, Ohio


### STERILITY IN WOMAN—ITS ÆTIOLOGY AND TREATMENT.

BY E. S. McKEE, M. D., CINCINNATI.

Your essayist first intended to write a paper on "Sterility in Woman." In a preliminary glance over the field he found he was undertaking a book. Having some regard for the feelings of his hearers, he limited the title to "Its Ætiology and Treatment."

The mariner wending his way over the wide ocean is accustomed now and then to take observations and to find what progress has been made, or how far, if any, he has drifted from the proper course. The oftener and the more carefully he makes these observations, the less will be his deviations and the sooner he reaches the desired haven. So with us is it wise from time to time to consider what we have done, even though the way we travel may be well worn and apparently barren and unfruitful.

If is only in rare instances that the lack of descendants is not sooner or later regretted by both husband and wife, and it often proves an opening of the door to marital discord and reproaches, with all their evil consequences. Sterility, among the old Romans and Israelites was sufficient cause for dissolution of the marriage relation. Instances of human sterility due to want of sexual harmony are exceedingly rare, and most cases supposed to be of this nature can be otherwise explained. The ætiology of primary sterility is often "shrouded in darkness," and the successful treatment of the same past finding out.

Sterility in man must necessarily be eliminated before we can be assured of success in treating the woman. A few years ago man was supposed to be able to beget children in almost every instance. Gross has proven otherwise. His statement that one case in six is due to sterility on the part of the man is probably too strong, yet it is not greatly wide of the mark. We can not believe, as we did a few years ago, that the paramount source of sterility is in the female. Kehrer, after a series of carefully conducted experiments, has arrived at the conclusion that "in at least a third of the cases of sterile marriages the husband was the party at fault, and gonorrhœa the cause of the barrenness."

The most frequent origin of sterility is, perhaps, intra-uterine disease, and chronic endometritis is its usual manifestation. This disease may act by giving rise to the characteristic profuse gelatinous discharge, thus hindering the ingress of spermatozoa by destroying the vitality of the spermatozoa. This renders the mucous membrane unfit for the fixation and development of the ovum. This diseased condition of the intra-uterine mucous membrane may extend into the Fallopian tubes and obliterate their orifice so as to prevent the admission of the spermatic fluid. It is well known that constitutional causes, more especially the scrofulous diathesis, have much to do with these inflammations; also cold and heat, overfeeding and underfeeding, youth and old age, lowering of general health, confinement and interbreeding.

Diligent study of the subject of sterility demonstrates that inflammations of the pelvic peritoneum, and of the parametria, or rather their consequences, are among the most frequent causes of sterility. Anatomical researches very frequently find such considerable adhesions between oviduct, ovary, uterus and rectum that conception would be an absolute impossibility, yet other adhesions exist which might not prevent impregnation.

Three questions are to be determined: (1) Are spermatozoa in the semen? (2) Do they get into the utero-cervical canal? (3) Do the secretions in the canal poison the spermatozoa?

Microscopical examinations were made by Roy, of Munich, of the condition of the spermatozoa at different intervals after coitus, in sixty women who were under treatment for sterility. In fifty-seven catarrh was present. In all of these cases only a small number of spermatozoa could be detected within the uterus, and they had all become motionless within five hours after coitus. In healthy women he had found that the movements of the spermatozoa within the uterus continued for at least thirty-six hours.

It is probable that if the secretions are normal the spermatozoa can make its way into the uterus in spite of flexions or stenoses. We learn from physiologists that the healthy spermatozoon in a normal vagina may live and retain its vitality for seven or eight days; that its lateral diameter is one forty-two hundredths of an inch, and that it will be able to penetrate any orifice through which a red blood corpuscle can pass. Its rate of travel is about seven and a half or eight inches in an hour, sixteen feet in a day, or thirty or forty yards in its lifetime. These facts would lead us to doubt if long or narrow or ante or retro-flexed cervices could primarily have anything to do with sterility.

Vulvar or vaginal hyperæstheria, inflammation of the carunculæ myrtiformes, undue shortness of the vagina, unless great care is exercised by the husband, will induce dispareunia and may also bring about sterility by favoring the formation of a copulative sac outside of the axis of the uterine canal, and consequent destruction of the semen.

Infantile uteri and other malformations are frequent causes. Malformations of the vaginal portion often prevent conception in women as well as animals.

A Chicago professor stated in a clinical lecture that it had been his observation that in sterile women the hair on the mons veneris was always straight. That curling the hair would cure the sterility was not demonstrated.

Premature and post-mature marriages seem to tend to sterility.

The infertility of heiresses is well known and goes to show sterility to a certain extent inherited, if inherited sterility is not a contradiction of terms. This is one way in which persons become rich—property increases, children are few.

The female being less passionate than the male the orgasm comes later with her. We can, with reason, suppose that conception is more liable to occur were it to happen simultaneously. The male orgasm occurring so early, the female may not reach that state at all.

Obesity is especially regarded as a potent factor in sterility, and the state of nutrition in woman, as in plants and animals, has long been known to have this tendency. Some have attempted to explain the connection between obesity and sterility by the direct pressure of the fat upon the ovaries.

The more plausable theory is that the extensive disposition of fat deducts from the developmental process in the organs. The interference with menstruation which we observe so often in fleshy persons points in this direction. It is probable that the ovaries are not organically affected by this condition, as the normal menstrual flow generally reappears when the obesity disappears. Examination of the ovaries, under chloroform, in obese persons, will show their organs intact, and it is probable that obesity causes sterility indirectly by hindering the expulsion of the ova and not their development.

The injurious influence of fatness in women, as regards child-bearing, is universally admitted, and is corroborated by experience with plants and the lower animals. Young women, upon commencing to breed, are fat, or at least plump. When they bear children they lose in weight by diminution of fat, and when they again cease to bear children they regain their fat with compound interest. This premature and post-mature fat is, to a certain extent, an indication of health.

When it attains a condition of polysarcia obesity has a great influence on generation. In the male, its early development checks the growth of the genital organs, and if late diminishes sexual desire. The accumulation of fat in the abdomen is claimed by some to exercise an injurious pressure on the utero-ovarian apparatus.

The hypertrophic condition of the external genitals in obesity may form a mechanical hindrance to impregnation. Given an obese woman, we will generally find that the prospect of offspring will depend more upon the menses than the amount of fat, amenorrhœic fat women being usually sterile. Statistics show a diminished increase in population during famine, but it is only ephemeral. Thinness only affects fertility when it is depends upon some chronic disease.

Over feeding and luxurious habits have not a little influence on sterility. It is a matter of common observation that the laboring classes, among whom destitution frequently prevails, are much more fertile than the more wealthy. The indolent and luxurious mode of life prevalent among the rich diminishes fertility, while this condition is favored by the laboring habits and diet of the poor. The proportionate fertility of the classes is stated by Marshall Hall to be six to one.

Venereal diseases have their share of influence, and the gonorrhœal affection is a potent cause of sterility, but it is by no means proven that syphilis has any unfavorable influence on conception, though the frequent abortions due to this or other causes may lead to this result. Syphilis is often associated with gonorrhœa, which is the real cause. Gonorrhœa frequently prevents conception by the inflammation traveling up the womb along the fallopian tubes and renders the covering of the ovary thick and tense, so that the ovum cannot escape, or, if it does, the fimbriated extremity of the tube is so agglutinated that it cannot rise up to grasp the ovum. The extreme views of Maggrath, that the wives of men who have had gonorrhœa, as a rule, remain sterile, have not been extensively accepted by the profession.

Reflux of semen after coition was described as a cause of sterility by Hyppocratus and Loranus, and is probably a frequent cause, though, doubtless, many women base their complaints on a delusion.

This *profluvium seminis* is rarely complained of except by the sterile and is unfrequent among the fertile. It is also believed to be common among those sterile women who have no sexual pleasure. It is probable that the mucous discharges of the glands of Cowper or Dunerway are in some cases mistaken for semen.

The vaginal secretions under certain pathological conditions become so acid that it induces sterility. Women who suffer from severe vaginal catarrh are frequently sterile; the spermatozoa being found dead in the vagina some hours after copulation, though on examination, a shorter time revealed them still alive. This vaginal secretion, which is the *materia peccans*, may also act in a mechanical way. In cases where conception occurs, despite a very acid condition of the vaginal secretion, it is probable that some of the spermatozoa enter the uterus before the secretion has time to act on it, or possibly the spermatozoa being injected in a mass, the acid secretion is unable to penetrate and kill all of the spermatozoa.

Chlorotic women often conceive and sterile women just as often show no other cause for this sterility than chlorosis. Scrofula, probably by its effect on the general system leading to a deficient development of the entire body, genitals included, may be productive of sterility. Tuberculosis is, probably in all except its later stages, without effect on child bearing.

Alcohol has been considered as having a great influence on sterility in women. It evidently does diminish sexual potency in the male, and for this reason is largely blamed on the female. By causing general constitutional disorder and a chronic inflammation of the ovaries, it possibly does occasion a certain amount of sterility. Yet we see numerous instances of parents addicted to the abuse of alcohol who have large families of (very ragged) children.

Carcinoma cervicis uteri is an important obstacle to conception. When the growth has advanced to any degree, even if confined to only one wall, the cervical canal is mechanically stopped and the corrosive fluid which accompanies ulceration has a deleterious effect upon the sperm.

The higher education of women has been held to be a feature in the production of sterility. However, the experiment is one of rather too recent date to be of much certain value.

Ovulation is doubtless more perfectly and frequently performed in some women than in others. Some conceive with more or less regularity every fifteen or eighteen months, and others at intervals of several years.

Dysmenorrhœa among the fertile is comparatively uncommon. Nearly half of sterile women suffer from dysmenorrhœa. The association of this neurosis with sterility is not unimportant. We often have also the return of semen and derangement of sexual orgasm or coitus and the cure of the dysmenorrhœa is a direct step toward the cure of sterility. Some claim that two out of five cases of sterility are accompanied by spasmodic dysmenorrhœa.

Emmet in his tables shows that of all married women who suffered pain during menstruation at puberty, seventy-one per cent. were sterile.

Sexual incompatibility is well known to exist, prominent examples being Angustus and Livia, Napoleon and Josephine. A case is reported by Duncan, where a man married successively three childless widows and had children by each one of them, and another, where a woman is married successively during child-bearing limits, to three men, and has children by but one of them. Sterility from sexual incompatibility, we cannot foresee or prevent, and religion, morals and law interdict the cure that might result from a change of husband. Among some classes in Wales and Scotland, for instance, custom permits and local morals do not condemn a practice which produces many illustrations of this mutual incompatibility. This practice is called bundling, or keeping company, and consists in permitting young girls to cohabit with an eligible man, on the understanding that, if pregnancy ensues, the legal marriage tie is made; progeny not resulting, the woman is deserted by her friend and falls to another with whom the result may be different.

Sexual incompatibility, or want of sexual unity, the so-called relation sterility, is the condition in which long and regular cohabitation between two individuals remain without result, though each one can pro-create with other individuals. Some marriages remain long sterile, are dissolved and both men and women produce children in another marriage. Duncan has found that many more women relatively who marry between the ages of fifteen or twenty are sterile than those who marry after twenty.

Sexual sensations, lack of participation or feeling, seems to have no especial influence on conception. Many women who have no passion conceive rapidly; others who have, may not conceive at all. Dyspareunia or frigidity does not occupy a very important role in sterility, though they do doubtless in some cases. Among prostitutes, the frequent delay of menstruation, then abundant hemorrhages are in many cases only habitual abortions and lead to changes in the genitals which must result in sterility,

The effect of nervous or psychic influence on sterility through its suppression of menstruation, as we cannot reject the idea of a connection between ovulation and menstruation, doubtless is of some considerable moment and merits further investigation.

The Druidic college of the 12th century considered tannin a most potent cause of sterility, hence excessive tea drinking might act in the same way. Sulphur is also believed to have the same effect.

Such dislocations of the cervix may occur that instead of lying in a pool of semen as it should, it lies above, in front, or away from it, and this may prevent conception. Sterility can be occasioned where necessary by obliterating the uterine extremities of the fallopian tubes with the thermo-cautery.

The universal prolificness of Arkansas is well known and if you ask down there why they have so many children, they will reply that the mosquitoes are so bad that they cannot sleep at night. That mosquitoes are a stimulant to reproduction is hardly proven, but that they act indirectly is firmly believed in Arkansas.

Montagazza, Bondin and Balley wrote that consanguineous marriages tended towards sterility. Darwin, after a most thorough investigation, using as his guide Burke's "Landed Gentry" and "Peerage," finds that consanguineous marriages are slightly more fertile than the non-consanguineous. He is of the opinion that this is because marriage between first cousins is more apt to take place where there is a large group of persons who bear that relationship to each other and this fertility becomes hereditary. The alleged infertility of consanguineous marriages cannot be substantiated.

